# Drinking and pleasure: Interdisciplinarity points the way forward

**DOI:** 10.1111/add.70065

**Published:** 2025-03-27

**Authors:** James Nicholls, Geoffrey Hunt

**Affiliations:** ^1^ Institute for Social Marketing and Health, Faculty of Health and Sports Science University of Stirling Stirling UK; ^2^ Centre for Alcohol and Drug Research Aarhus University Aarhus Denmark; ^3^ Institute for Scientific Analysis Alameda California USA

**Keywords:** alcohol, epidemiology, interdisciplinarity, pleasure, psychology, sociology

*There is an appetite for further research on alcohol and pleasure, focusing on both theoretical and practical considerations. The complexity of the issues involved means such research will, by necessity, involve interdisciplinary collaborations. The responses to our article highlight several promising directions this work could take*.

We thank our commentators for their thoughtful reflections on how alcohol research can better engage with pleasure. In writing the original article [1], we admit to some trepidation about how it would be received. We are encouraged by the positive and constructive responses, which identify a range of opportunities for innovative future research.

We strongly agree with Pennay and Livingston [2] on the critical importance of interdisciplinary collaboration. Drinking motives, cultures, behaviours and pleasures – as well as risks – are far too complex to be captured by either a single discipline or a single methodological approach. The social, natural and applied sciences, and, we would add, the humanities, all offer unique contributions to better understanding the protean role of drinking in both different cultures and the lives of individuals within those environments. We would welcome the kind of large‐scale interdisciplinary project Pennay and Livingston propose, and hope the case for such an approach, even if exploratory in terms of both methods and possible findings, can be made effectively to funders.

Morris and Davies [3] provide critical insights into the challenges of effective alcohol health messaging. While we noted that negative framings may not align with the experiences of those who drink for pleasure, they expand significantly on this with key insights from experimental psychology. As they show, the issue is not only that ‘no safe level’ messaging may fail to resonate with drinkers, but that it may provoke psychological resistance – especially among heavier drinkers, who may be the primary target.

Acuff and Strickland [4] take a different approach, arguing not only for interventions that acknowledge the pleasures of drinking (even while encouraging less risky behaviours), but also for those that promote alternative pleasures not involving, though perhaps adjacent to, intoxication. This notion has deep historical roots, from the 19th century ‘rational recreation’ campaigns to the contemporary ‘sober curious’ movement [5, 6]. They also highlight key experimental research on wider determinants of alcohol‐related reward, which we would see as complementing sociological studies on the social structuring of intoxication and pleasure.

These commentaries helpfully expand on the three domains highlighted in our article. They speak to the need for a broader epistemology of intoxication, one that will necessarily be interdisciplinary. They also add psychological depth to our thoughts on the pragmatics of health communication. Furthermore, they highlight that a rigid prioritisation of long‐term health over shorter‐term rewards cannot survive contact with the reality of how and why people drink. Acuff and Strickland's compromise – acknowledging but not emphasising pleasure – is attractive. However, we would also reiterate our challenge for clarity on questions including why, even from a utilitarian perspective, intoxication per se is an ethical problem. These are the types of assumptions that could be more explicitly articulated, particularly when justifying potentially restrictive interventions that impact large populations. They are also the kinds of questions that could be explored in future interdisciplinary research, and we are pleased to find this is something colleagues are eager to pursue.

## AUTHOR CONTRIBUTIONS

**James Nicholls:** Conceptualization; writing—original draft; writing—review and editing. **Geoffrey Hunt:** Conceptualization; writing—review and editing.

## DECLARATION OF INTERESTS

None.

## FUNDING INFORMATION

None.

## Data Availability

Data sharing is not applicable to this article as no new data were created or analysed in this study.
